# Delayed treatment with ceftriaxone reverses the enhanced sensitivity of TBI mice to chemically-induced seizures

**DOI:** 10.1371/journal.pone.0288363

**Published:** 2023-07-13

**Authors:** Simone A. A. Romariz, Bevan S. Main, Alex C. Harvey, Beatriz M. Longo, Mark P. Burns

**Affiliations:** 1 Laboratory for Brain Injury and Dementia, Department of Neuroscience, Georgetown University Medical Center, Washington, DC, United States of America; 2 Departamento de Fisiologia, Laboratório de Neurofisiologia, Universidade Federal de São Paulo, UNIFESP, São Paulo, Brazil; Belgrade University Faculty of Medicine, SERBIA

## Abstract

The pathophysiological changes that occur after traumatic brain injury (TBI) can lead to the development of post-traumatic epilepsy, a life-long complication of brain trauma. The etiology of post-traumatic epilepsy remains unknown, but TBI brains exhibit an abnormal excitatory / inhibitory balance. In this study, we examine how brain injury alters susceptibility to chemically-induced seizures in C57Bl/6J mice, and if pharmacological enhancement of glutamate transporters can reduce chronic post-traumatic seizures. We found that controlled cortical impact (CCI) mice display delayed susceptibility to pentylenetetrazol (PTZ)-induced seizures. While CCI mice have no change in seizure susceptibility at 7d post-injury (dpi), at 70dpi they have reduced latency to PTZ-induced seizure onset, higher seizure frequency and longer seizure duration. Quantification of glutamate transporter mRNA showed that levels of *Scl1a2* and *Scl1a3* mRNA were increased at 7dpi, but significantly decreased at 70dpi. To test if increased levels of glutamate transporters can ameliorate delayed-onset seizure susceptibility in TBI mice, we exposed a new cohort of mice to CCI and administered ceftriaxone (200mg/kg/day) for 14d from 55-70dpi. We found that ceftriaxone significantly increased *Scl1a2* and *Scl1a3* in CCI mouse brain at 70dpi, and prevented the susceptibility of CCI mice to PTZ-induced seizures. This study demonstrates cortical impact can induce a delayed-onset seizure phenotype in mice. Delayed (55dpi) ceftriaxone treatment enhances glutamate transporter mRNA in the CCI brain, and reduces PTZ-induced seizures in CCI mice.

## Introduction

The consequences of traumatic brain injury (TBI) include pathophysiological changes, triggered at the moment of the primary injury, that extend for days, weeks or months after the event. This process of secondary injury includes inflammation, synaptic remodeling, astrogliosis, cell proliferation and autoimmune responses. The chronic consequences of TBI include cognitive [1–4] and emotional [3, 5] deficits, and in some cases can even result in post-traumatic epilepsy (PTE) with the development of epileptic seizures [6]. PTE is an important life-long complication of TBI and accounts for 10–20% of symptomatic epilepsies and 5% of all types of epilepsy [7]. Chronic PTE is considered difficult to treat, as it does not respond to common anti-epileptic drugs [8, 9]. The underlying causes of PTE remain under investigation, but an imbalance of the excitatory / inhibitory balance of the brain is thought to be involved.

Glutamate is the major excitatory neurotransmitter of the central nervous system, playing a crucial role in synaptic plasticity, learning and memory. The control of extracellular glutamate levels is important for the proper function of synapses and to avoid excitotoxicity. An excess of extracellular glutamate generates excitotoxic neuronal death that is associated with several neurological diseases such as Alzheimer’s disease, ischemia, TBI and epilepsy [10, 11]. Glutamate transporters are responsible for uptake glutamate from the synaptic cleft to maintain glutamate homeostasis and prevent excitotoxic neuronal death. Excitatory amino acid transporters 1 (EAAT1) and 2 (EAAT2) are primary glutamate transporters in the central nervous system (CNS) in humans and their equivalents in rodents are glutamate aspartate transporter (GLAST), encoded by Slc1a3 gene and glutamate transporter 1 (GLT1), encoded by Slc1a2 gene. These glutamate transporters are principally expressed in glial cells and GLT1 accounts for approximately 90% of synaptic glutamate clearance [12, 13]. Dysfunction of glutamate transporters can influence diseases such as epilepsy and TBI. In post-mortem TBI brains, EAAT2 appears to be increased with 24h of injury, and decreased after 24h of injury [14]. Experimental studies have showed an acute reduction of GLT1 expression after TBI and rats with GLT1 expression deficits have increased neuronal death when exposed to TBI [15, 16]. Therefore, therapeutic strategies that increase or regulate the glutamate transporters expression could be a promising approach for the treatment of PTE.

The use of rodent models of TBI allow for the systematic study of the mechanisms that underlie PTE. Controlled cortical impact (CCI) is a well-characterized model of focal TBI with a contusion injury, secondary injury, and a consistent neuropathological response. The existence of spontaneous seizures remain controversial in the field, however multiple groups have reported EEG evidence of spontaneous seizures in young and adult rodents at both the early-stage post-TBI [17–19] and late-stage post-TBI [18, 20–22]. CCI also causes increased seizure susceptibility to a chemical convulsant (pentylenetetrazol (PTZ)) [20, 23, 24], demonstrating that the threshold for seizure formation is lower in TBI animals compared to sham controls.

Our aim in the present study is to study seizure susceptibly in CCI mice during two distinct windows after injury–a subacute (7 days post-injury) and chronic (70 days post-injury) phase. Furthermore, we quantified the expression of GLT1 and GLAST mRNA from the cortex and hippocampus of CCI mice, and examined the ability of the β-lactam antibiotic ceftriaxone (CEF) to enhance GLT1 and GLAST mRNA, and reduce seizure susceptibility in the TBI brain when administered in the chronic phase after injury.

## Methods

### Animals

All experimental procedures were approved by the Georgetown University Animal Care and Use Committee (Protocol #2016–1165). Male C57Bl/6J mice (Jackson Labs, Bar Harbor, ME) were housed 5 per cage under 12:12 h light:dark cycles with water and food *ad libitum*, and maintained at a temperature of 18-24C and 40–60% humidity. Mice were 10–12 weeks of age at time of injury.

### Controlled cortical impact (CCI) mouse model

CCI was performed as previously published [25, 26]. CCI injury was induced with a Leica Impact One Stereotaxic Impactor device. Mice were anesthetized with isoflurane (induction at 4%, maintenance at 1.5% in 1.5L/min oxygen). Mice were then placed on a stereotaxic frame with built in heating bed to maintain body temperature at 37C for the duration of the surgery. The mouse’s head was secured in the stereotaxic frame and the surgical site was clipped and sanitized with iodine and ethanol pads. Bupivacaine was administered as an analgesic at the surgical site, and a 10 mm midline scalp incision was made, followed by a 4 mm craniotomy over the center of the left parietal bone (-2.0mm A/P, -2.2mm ML). The 3.5mm steel impactor tip was slowly lowered to contact the exposed dura at the craniotomy site, then the tip was retracted and set to the desired injury depth. The impact occurred with a velocity of 5.25 m/s, a 100ms dwell time and a 1.5 mm injury depth. After the injury, the incision was closed using wound clips, the animal was removed from anesthesia and placed on a heating pad for recovery. Sham injury consisted of anesthesia and the midline incision, with no craniotomy or impact. Wound clips were removed 10–14 days post-surgery.

### T-Maze

We tested spontaneous alternation in CCI mice using a mouse T-maze (San Diego Instruments) as previously reported [27]. Mice were placed in the start arm and allowed to acclimatize for 30 seconds. The start door was opened and mice were allowed to ambulate up the starting arm and make a choice of the left or right arm. Once inside the chosen arm, the door to the arm was closed and the mouse confined in the chosen arm for 30 seconds. Mice were then removed the maze and returned to their cage and the test was then repeated within 5 minutes. This process was repeated 3 times on each mouse, with at least 1h between each session. The number of times that the mice chose the novel arm during the second phase of the trial (spontaneous alternation) was recorded. All testing was performed and analyzed blind to condition.

### PTZ-induced seizures

PTZ was administered by intraperitoneal injection at either 30mg/kg or 60 mg/kg as indicated in the text. Immediately following PTZ administration, sham and CCI mice were monitored for 30 minutes and their seizures recorded by an observer blinded to treatment condition. Seizures were scored using a revised Racine scale [28], as show in Table 1. Only behavioral seizures that are clearly identified (i.e. Score 3–7) were included in the analysis, with type 3 and 4 assigned as a low-severity seizure and 5–7 assigned as high-severity seizures. The following criteria were analyzed: latency to seizure onset, seizure frequency, seizure duration (the total duration of all seizures in a mouse), seizure severity, and seizure mortality.

**Table 1:** Modified Racine score for PTZ-induced seizures. From [28].

Modified Racine Score	Behavioral Correlate
-1	Normal baseline
0	Whisker trembling
1	Sudden behavioral arrest
2	Facial jerking
3	Neck jerks
4	Clonic seizure (sitting)
5	Clonic, clonic-tonic seizure (lying on belly)
6	Clonic, clonic-tonic seizure (lying on side), wild jumping
7	Tonic extension, possibly leading to death

### Ceftriaxone treatment

CCI and sham mice were randomly divided into experimental groups. Ceftriaxone mice were administered drug in 0.89% saline at a concentration of 200mg/kg/day, delivered once per day by intraperitoneal injection. Vehicle mice received saline alone. Drug treatment started 56d after CCI injury, with the final injection administered 2h prior to PTZ challenge. 5-7d of ceftriaxone (200 mg/kg) has previously been shown to increase GLT1 protein expression in the hippocampus of mice [29]. As we did not observe any seizures in vehicle sham mice challenged with 30 mg/kg PTZ, we combined this group with the sham ceftriaxone group together for the final analysis.

### Euthanasia

Mice were euthanized 3h after the PTZ challenge by CO2 euthanasia followed by cardiac perfusion with PBS. Brains were extracted and a 7.5mm punch from the pericontusional area of the ipsilateral cortex was removed, as well as the ipsilateral hippocampus were dissected. Whole ipsilateral hippocampus and ipsilateral cortical punch tissue were used for RNA analysis.

### RNA isolation, cDNA synthesis, real time quantitative polymerase chain reaction (RT-QPCR)

RNA was extracted from brain tissue using TRIzol^®^ reagent (15596026, Invitrogen). Concentration and purity of the RNA was assessed using a NanoDrop 1000 spectrophotometer (ThermoScientific) as previously described [25, 30]. One microgram of RNA was reverse transcribed into cDNA using a High-Capacity RNA-to-cDNA Reverse Transcription Kit (4368814, Applied Biosystems, Foster City, CA). The resulting cDNA was diluted 1:3 with diethylpyrocarbonate (DEPC) treated H_2_O for use in RT-QPCR.

RT-QPCR was performed in triplicate in standard 384-well plates (4309849, Applied Biosystems) using the Prism 7900HT fast sequence detection system (Applied Biosystems). The following Taqman probes were used: *GAPDH* (Mm99999915_m1), *Ubc* (Mm02525934_g1), *Gfap* (Mm01253033_m1), *Slc1a2* (Mm01275814_m1),*Slc1a3* (Mm00600697_m1) and analyzed under the following cycle conditions; 50°C for 2 minutes, 95°C for 20seconds, (95°C for 1 second, 60°C for 20 seconds) with 40 cycles. SDS 2.4 software (Applied Biosystems) was used to generate threshold cycle (Ct) values, and fold change in mRNA expression calculated using the ΔΔCt method (2^-ΔΔCt^).

### Lesion quantification

Lesion quantification as previously described [26]. PBS-perfused brains were drop-fixed in 4% paraformaldehyde. Brains were sliced into 30 μm-thick coronal section, and stained with cresyl violet (0.1%) in water for lesion quantification. Slides were imaged (9 brain sections through the lesion site for each mouse), and the lesion was quantified using Image J. Percent tissue loss was calculated by dividing the area of the ipsilateral tissue by the area of the contralateral hemisphere x 100.

### Statistics

All data are presented as mean ±S.E.M. Datasets comparing only two groups were analyzed using unpaired, two-tailed t-tests. Datasets with more than two groups were analyzed either using a one-way ANOVA with Tukey’s multiple comparison’s post-hoc test for one factor designs, or using a two-way ANOVA with Tukey’s multiple comparison’s post-hoc test for two-factor designs. Survival data was analyzed using a Chi-squared test. The criteria for statistical significance was preset at p<0.05 for all experiments. All statistical analysis was performed using Graph Pad Prism™ Software (version 8.0). Group size for each experiment as follows: PTZ in sham and CCI mice n = 14 per group; PTZ in ceftriaxone and vehicle mice = 9–18 per group; mRNA analysis for sham vs CCI n = 10; mRNA for ceftriaxone and vehicle n = 6–12; Lesion quantification in untreated CCI mice n = 3–4.

## Results

### CCI causes impaired working memory deficits at both sub-acute and chronic timepoints post-TBI

**Fig 1:**
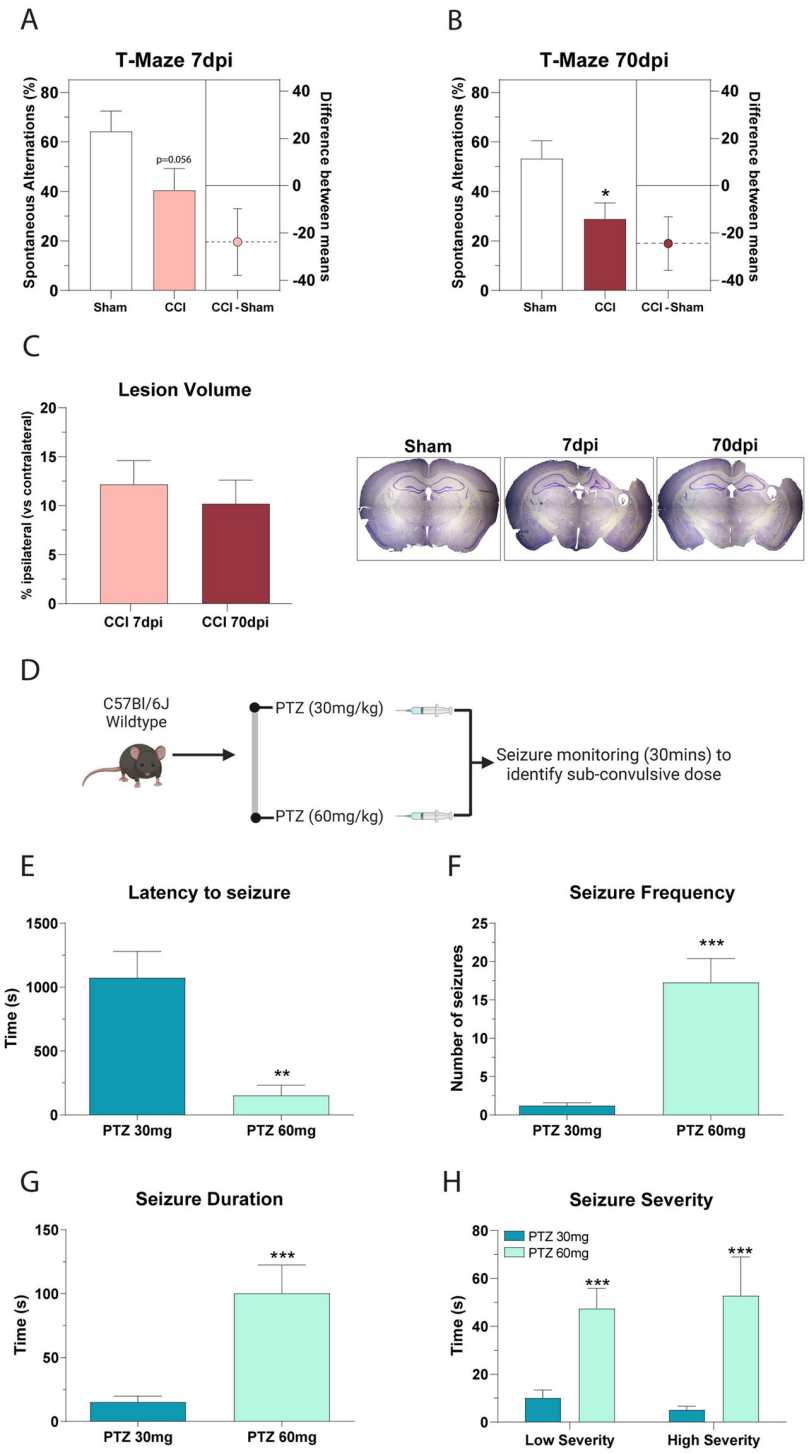
Characterization of the controlled cortical impact model and selection of sub-convulsive dose of PTZ. **A, B)** CCI mice at 7dpi and 70dpi have similar deficits in working memory. Spontaneous alternations were detected using the T-maze with CCI mice displaying a 24-point drop in working memory score at both timepoints. Unpaired t-test, *P = 0.016; n = 14. **C)** CCI mice have a similar loss in ipsilateral brain volume at 7dpi and 70dpi. n = 3–4 **D)** Schematic of the experimental design to select a sub-convulsive dose of PTZ. Seizure parameters were quantified following 30/mg/kg or 60mg/kg PTZ into naïve mice. Mice receiving 60mg/kg PTZ had **E)** a faster time to seizure onset, **F)** an increase in the number of seizures recorded and **G)** an increase in the average seizure duration compared to mice receiving 30mg/kg PTZ. 60mg/kg PTZ mice also recorded higher time in both low severity and high severity seizures. Unpaired t-test, ** = P < 0.01; *** = P < 0.001 n = 14 for 30mg/kg and 7 for 60mg/kg.

The T-maze was conducted at 7 and 70 days after TBI to test for working memory dysfunction. Alternation scores above 50% are considered to be above the rate of chance. At 7 days post injury (dpi), we found that sham mice had a spontaneous alternation rate of 64%, but CCI mice alternated at a rate of 40% (mean reduction of 23.8; P = 0.056, n.s.) (Fig 1A). A separate cohort of mice was used to test for chronic deficits at 70dpi, and we found that sham mice had a 53% rate of spontaneous alternations compared to only 29% in CCI mice (mean reduction of 24.4; p = 0.016) (Fig 1B). These data show that CCI mice have a similar reduction in working memory at 7dpi and 70dpi.

### CCI causes tissue loss at both sub-acute and chronic timepoints post-TBI

We quantified the lesion volume caused by CCI in both the acute and chronic phase of TBI. We found that tissue loss in the ipsilateral hemisphere compared to the contralateral hemisphere was 12% at 7dpi, and 10% at 70dpi. There was no significant difference in lesion volume between the sub-acute and chronic phase of CCI (Fig 1C).

### Identification of a sub-convulsive dose of PTZ for use in CCI mice

In order to identify a sub-convulsive dose of PTZ for use in CCI mice, we tested two doses of PTZ in naïve mice and examined the onset of seizures, and seizure strength. We found that while 60mg/kg PTZ caused a rapid onset of prolonged and frequent seizures, 30mg/kg had a minimal effect on seizure onset and seizure type (Fig 1D–1H). We concluded that 30mg/kg was an appropriate sub-convulsive dose for use in our experiments.

### CCI causes an increase in seizure susceptibility at chronic, but not sub-acute, timepoints post-TBI

**Fig 2:**
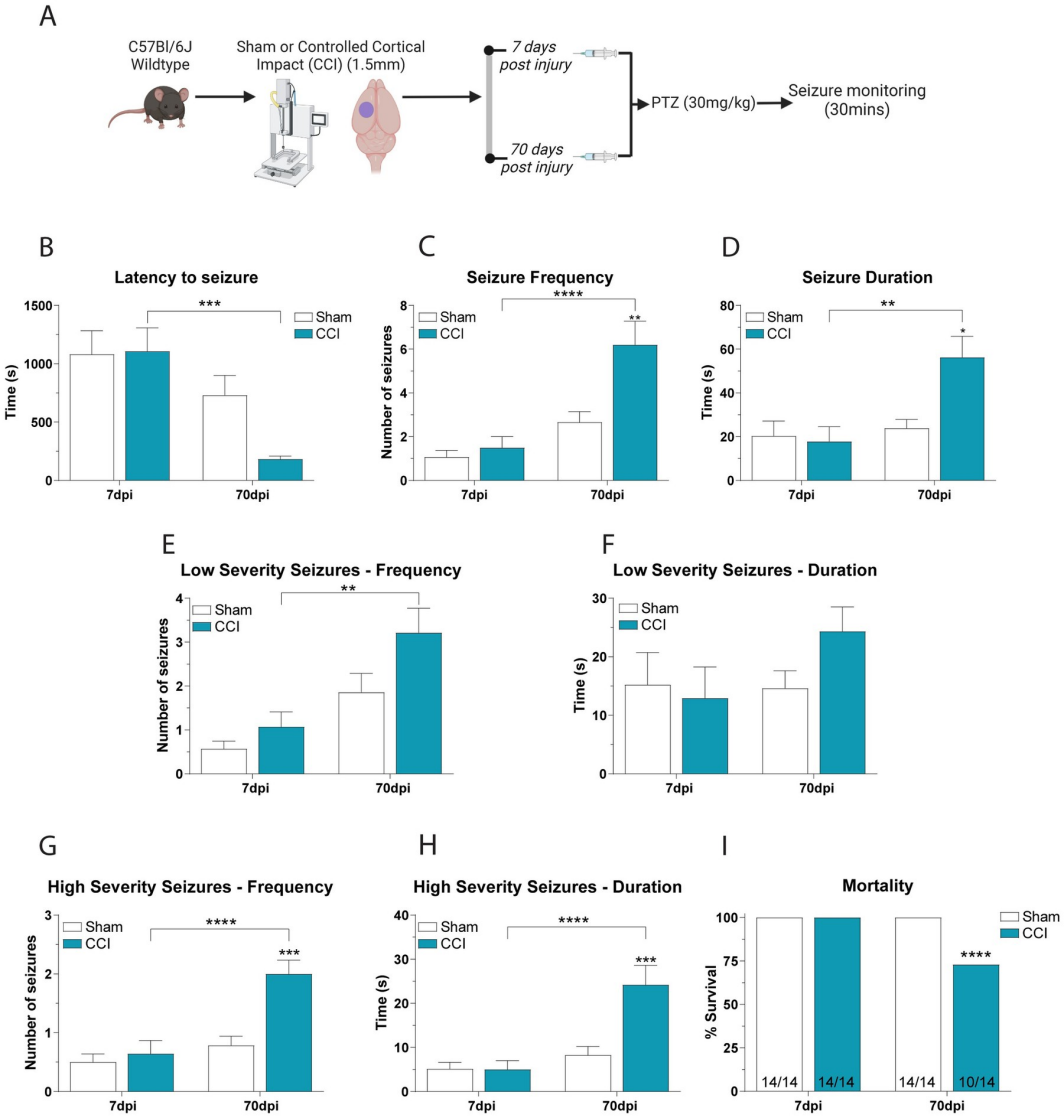
Chronic, but not acute, CCI mice have increased susceptibility to 30mg/kg PTZ. **A)** Schematic of the experimental design for this figure. Separate cohorts of CCI and sham mice were injected with 30mg/kg PTZ at either 7dpi or 70dpi. 70dpi mice had increased susceptibility to PTZ-induced seizures with **B)** reduced latency to seizure onset, **C)** increased seizure frequency, **D)** increased seizure duration, **E-H)** increased frequency and duration of low and high severity seizures, and **I)** increased mortality compared to CCI mice at 7dpi or sham mice. 2 Way ANOVA with Tukey’s multiple comparison test. * = P < 0.05, ** = P < 0.01; *** = P < 0.0001, **** = P < 0.0001 Mortality analysis using Chi-square test, **** = P < 0.0001 n = 14.

We exposed separate cohorts of sham and CCI mice to a sub-convulsive dose of 30 mg/kg PTZ at 7dpi and 70dpi (Fig 2A). We found that CCI only impacted PTZ-induced seizures at 70dpi and not 7 dpi. Analysis of latency of seizure onset revealed a significant effect of time (F_(1,52)_ = 17.58; P = 0.0001) with post-hoc analysis revealing an 85% reduction in latency between 7dpi CCI mice and 70dpi CCI mice (Fig 2B). For PTZ-induced seizure frequency, we found a significant effect of time (F_(1,52)_ = 22.52; P < 0.0001), a significant effect of injury (F_(1,52)_ = 7.76; P = 0.0074) and a significant interaction between the two variables (F_(1,52)_ = 4.693; P = 0.0349). Post-hoc analysis revealed a 319% increase in seizure frequency between 7dpi CCI mice and 70dpi CCI mice (P < 0.0001) and a 120% increase between 70dpi sham mice and 70dpi CCI mice (P = 0.005) (Fig 2C). Finally, for PTZ-induced seizure duration we also found a significantly effect of time (F_(1,52)_ = 8.966; P = 0.0042), and a significant interaction between time and injury (F_(1,52)_ = 5.198; P = 0.0267). Post-hoc analysis revealed a 215% increase in seizure duration between 7dpi CCI mice and 70dpi CCI mice (P = 0.0026) and a 120% increase in duration between 70dpi sham mice and 70dpi CCI mice (P = 0.0219) (Fig 2D).

In addition to seizure timing, we also found a significant difference in seizure severity, with CCI causing increased seizure severity at 70dpi, but not 7dpi (Fig 2E–2H). There was a significant effect on the frequency of PTZ-induced high-severity seizures, with a significant effect of time (F_(1,54)_ = 18.15; P < 0.0001), a significant effect of injury (F_(1,54)_ = 12.38; P = 0.0009) and a significant interaction between the two variables (F_(1,54)_ = 7.718; P = 0.0076). Post-hoc analysis revealed a 212% increase in high-severity seizure frequency between 7dpi CCI mice and 70dpi CCI mice (P < 0.0001) and a 156% increase in frequency between 70dpi sham mice and 70dpi CCI mice (P = 0.0003) (Fig 2G). There was a significant effect of time on PTZ-induced high-severity seizure duration (F_(1,54)_ = 17.01; P = 0.0001), a significant effect of CCI injury (F_(1,54)_ = 8.479; P = 0.0053) and a significant interaction between the two variables (F_(1,54)_ = 8.788; P = 0.0046). Post-hoc analysis revealed a 384% increase in high-severity seizure duration between 7dpi CCI mice and 70dpi CCI mice (P < 0.0001) and a 195% increase duration between 70dpi sham mice and 70dpi CCI mice (P = 0.0007) (Fig 2H). We also found that 29% of 70dpi CCI mice died following PTZ-induced seizure (4 of 14 mice), but no 7dpi CCI mice or sham mice died following PTZ (P < 0.0001) (Fig 2I).

### Changes in seizure susceptibility are associated with changes in glutamate transporter expression

Astrocytic glutamate transporters are responsible for controlling extracellular glutamate levels at the synapse, and dysfunction in these receptors is associated with changes in neuronal excitability [31, 32]. Given the time-dependent changes in seizure susceptibility following CCI, we compared mRNA expression of GLT1 transporter Slc1a2 and the GLAST transporter Slc1a3 from cortex and hippocampus at sub-acute and chronic timepoints post-injury.

**Fig 3:**
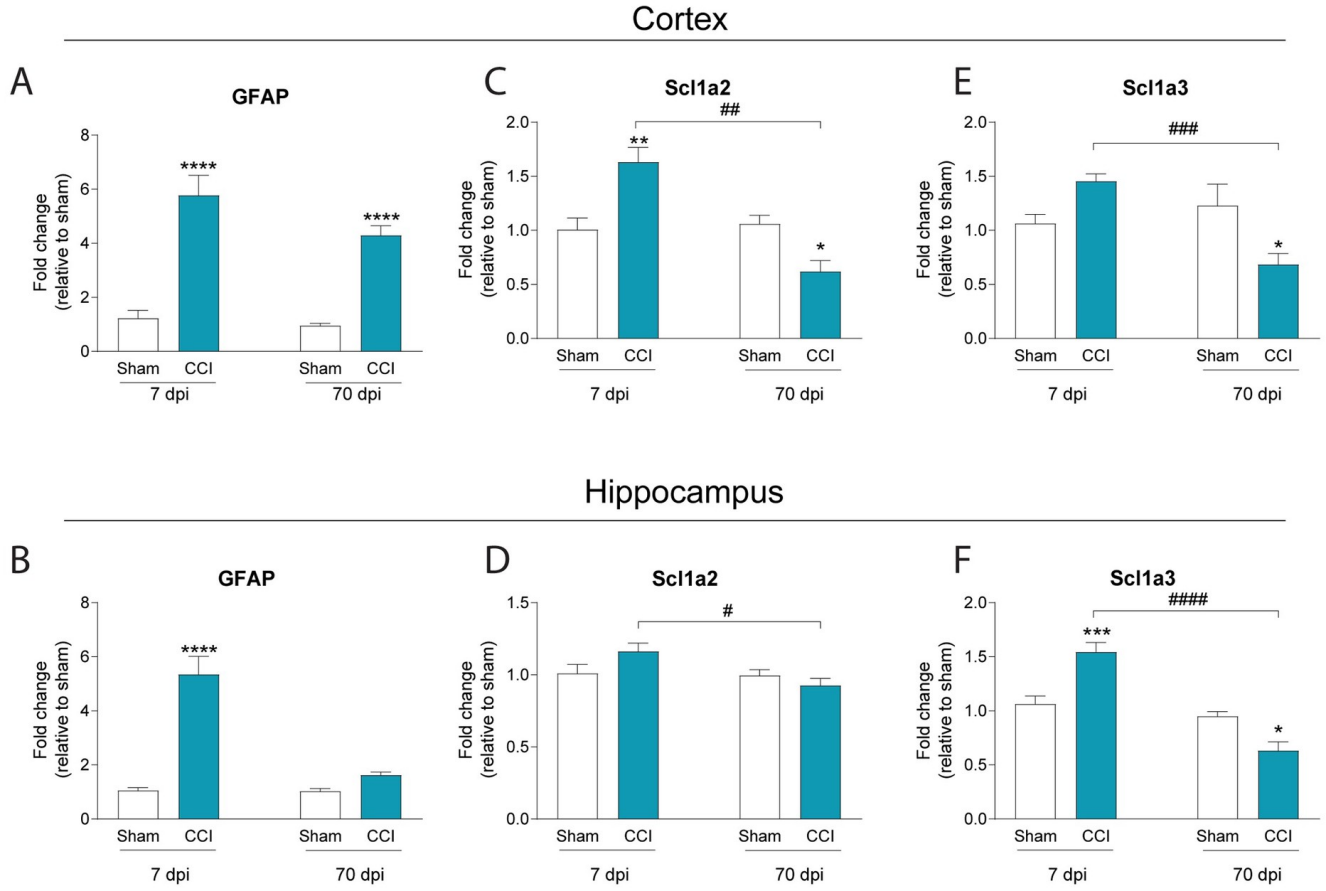
Time-dependent effects of CCI on glutamate transporter mRNA. **A, B)** CCI mice have increased GFAP mRNA in the cortex and hippocampus at 7dpi and in the cortex at 70dpi. **C, D)** CCI mice have increased Scl1a2 (GLT-1) mRNA in the cortex at 7dpi, but less Scl1a2 at 70dpi. Levels of Scl1a2 in the hippocampus are not different from shams at either timepoint. **E, F)** CCI mice have increased levels of Scl1a3 in the hippocampus at 7dpi, but reduced levels of mRNA in the cortex and hippocampus at 70dpi. 2 Way ANOVA with Tukey’s multiple comparison test. * = P < 0.05; ** = P < 0.01; *** = P < 0.001; **** = P < 0.0001. n = 10 for sham 7dpi, CCI 7dpi, sham 70dpi and n = 11 for CCI 70dpi.

We measured GFAP mRNA in the cortex and hippocampus. We found that there was a significant effect of CCI injury in the cortex (F_(1,38)_ = 82.49; P < 0.0001) and hippocampus (F_(1,38)_ = 54.68; P < 0.0001). In the cortex there was also a significant effect of time (F_(1,38)_ = 32.1; P < 0.0001) and an interaction between the two variables (F_(1,38)_ = 31.22; P < 0.0001). Posthoc analysis revealed that CCI increased GFAP at 7dpi in both the cortex (P < 0.0001) and hippocampus (P < 0.0001) and in the cortex at 70dpi (P < 0.0001) (Fig 3A and 3B).

We found that Scl1a2 expression was significantly altered in the cortex. Two-way ANOVA revealed a significant effect of Time (F_(1,38)_ = 19.9; P < 0.0001) and an time x injury interaction (F_(1,38)_ = 24.58; P < 0.0001). Post-hoc analysis revealed that Scl1a2 mRNA expression was significantly increased by 63% in the 7dpi CCI mouse cortex compared to sham (P = 0.0015), however it was significantly decreased by 32% in the 70dpi CCI mouse cortex (P = 0.0251) (Fig 3C and 3D). For Scl1a3 expression in the cortex, two-way ANOVA revealed a significant effect of Time (F_(1,38)_ = 6.138; P = 0.0178) and an time x injury interaction (F_(1,38)_ = 14.66; P = 0.0005). Post-hoc analysis revealed that Scl1a3 mRNA expression was reduced by 21% in the 70dpi CCI mouse cortex compared to sham (P = 0.0137), and reduced by 53% compared to 7dpi CCI cortex (P = 0.0003) (Fig 3E and 3F).

### Delayed ceftriaxone treatment increases Scl1a2 and Scl1a3 mRNA expression in the cortex and hippocampus of CCI mice

**Fig 4:**
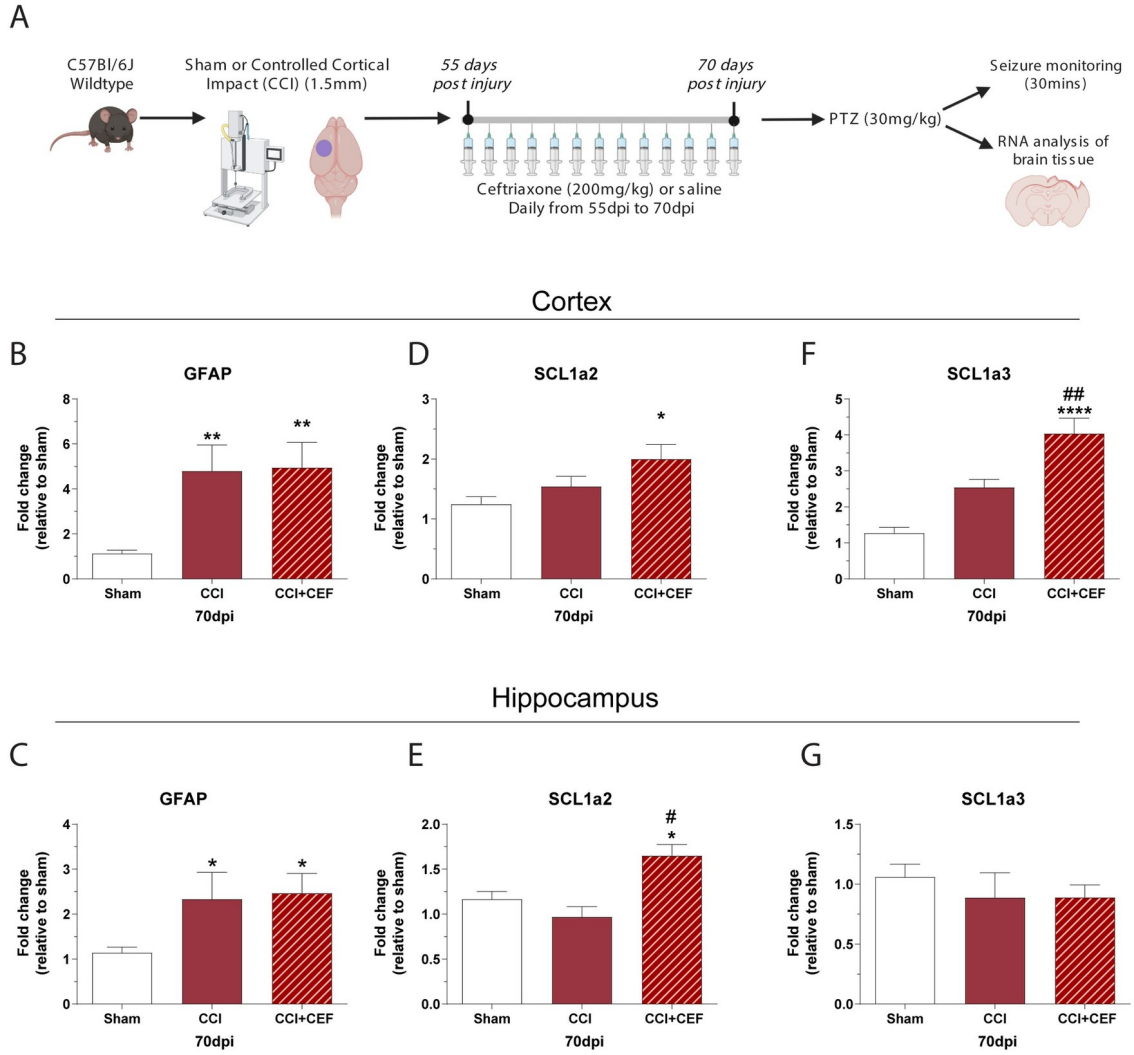
Delayed treatment with ceftriaxone increases glutamate transporter mRNA in cortex and hippocampus. **A)** Schematic of the experimental design. CCI or sham mice were injected with ceftriaxone (CEF) from 56dpi to 70dpi, then challenged with PTZ before tissue analysis for glutamate transporters. **B, C)** CCI resulted in an increase in GFAP in both the cortex and hippocampus that was not affected by ceftriaxone treatment. **D, E)** Ceftriaxone-treated CCI mice had a significant increase in SCL1a2 (GLT-1) mRNA in both the cortex and hippocampus. **F, G)** Ceftriaxone-treated CCI mice had significantly increased SCL1a3 (GLAST) mRNA in the cortex, but not in the hippocampus. ANOVA with Tukey’s multiple comparison test.* = P < 0.05; ** = P < 0.01; **** = P < 0.0001 vs sham. # = P < 0.05, ## = P < 0.01 vs CCI-vehicle. n = 12 for sham, 6 for CCI-veh and 6 for CCI-CEF.

We hypothesized that increased seizure susceptibility was due to a decrease in Scl1a2 and Scl1a3 in the cortex and hippocampus of CCI mice at 70dpi. We therefore treated mice with the antibiotic ceftriaxone, which has previously been shown to increase glutamate transporters in rodent brain [29]. In this experiment, we delayed treatment with ceftriaxone until 56dpi to allow the seizure-susceptibility phenotype to develop in our CCI mice. Ceftriaxone treatment (200mg/kg/day) was continued from 56dpi to 70dpi (Fig 4A).

There was a significant effect of TBI on GFAP mRNA in the cortex at 70dpi (F _(2, 21)_ = 10.94; P = 0.0006). Ceftriaxone treatment did not affect the TBI-induced increase in GFAP mRNA in the cortex or hippocampus of CCI mice, which was elevated by 424% in CCI vehicle treated cortex (P = 0.0036), and by 436% in the ceftriaxone CCI cortex (P = 0.0025) (Fig 4B). CCI also affected GFAP mRNA in the hippocampus (F _(2, 21)_ = 5.486; P = 0.0121). In the hippocampus GFAP mRNA was elevated by 133% in CCI vehicle treated cortex (P = 0.047), and by 147% in the ceftriaxone CCI cortex (P = 0.025) (Fig 4C). Scl1a2 mRNA was significantly affected by injury (F _(2, 21)_ = 5.031; P = 0.0164). Ceftriaxone significantly increased Scl1a2 mRNA by 60% in the cortex compared to sham (P = 0.0124) (Fig 4D), and by 66% in the hippocampus compared to sham (P 0.01) and by 115% compared to CCI vehicle mice (P = 0.0018) (F _(2, 21)_ = 8.686; P = 0.0018) (Fig 4E). In addition, ceftriaxone treatment increased Scl1a3 mRNA in the cortex by 217% compared to sham mice (P < 0.0001) (Fig 4F) and by 60% compared to CCI vehicle mice (P = 0.0037) (F _(2, 21)_ = 31.62; P <0.0001) (Fig 4G). We conclude that delayed ceftriaxone treatment can increase glutamate transporter mRNA in CCI mice without altering GFAP mRNA.

### Delayed ceftriaxone treatment reduces seizure severity in chronic CCI mice

**Fig 5:**
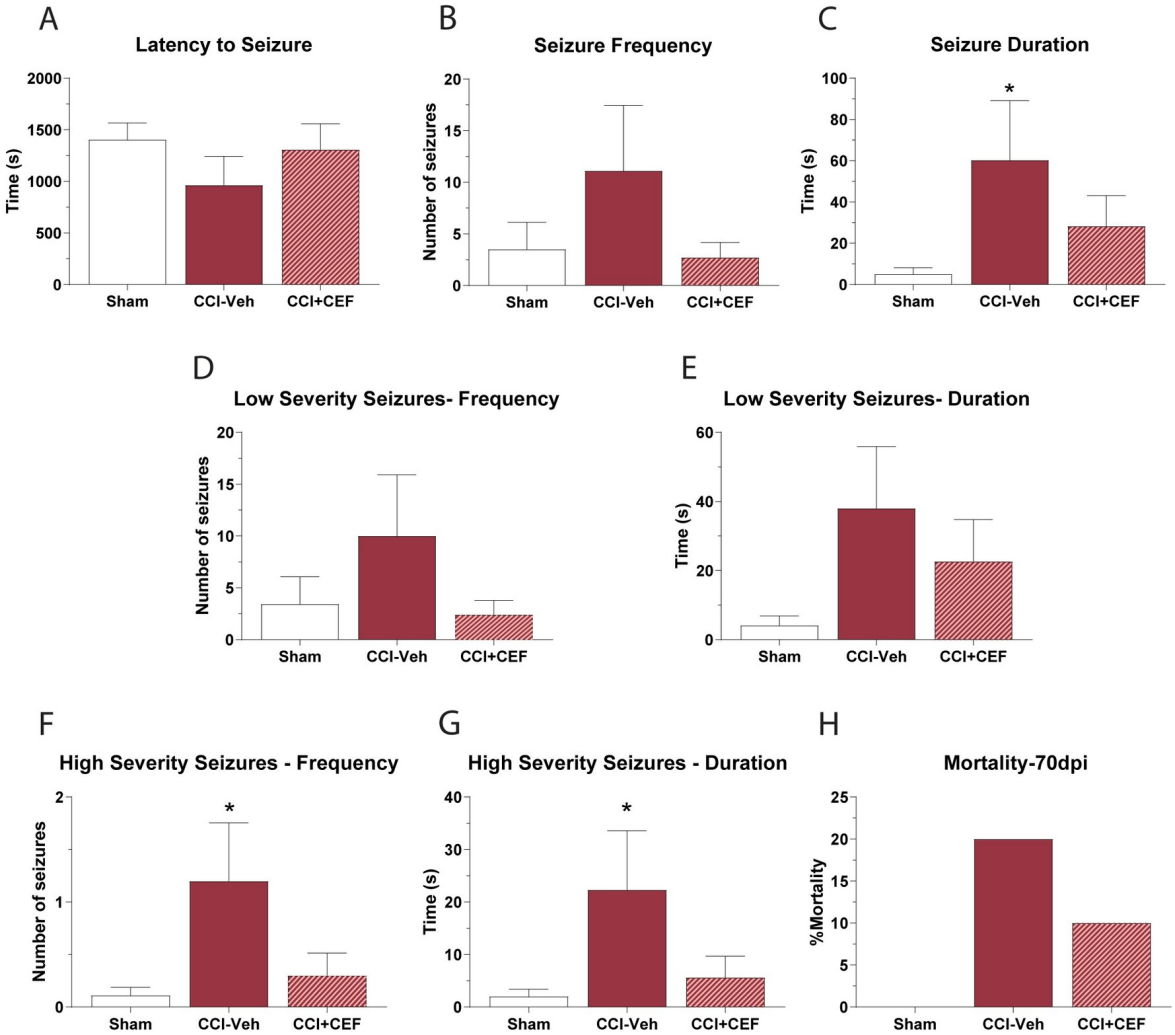
Delayed treatment with ceftriaxone reduced seizure susceptibility in CCI mice. CCI mice were injected with ceftriaxone (CEF) or vehicle from 56dpi to 70dpi. At 70dpi sham and CCI mice were challenged with 30mg/kg PTZ and seizure characteristics were assessed including **A)** latency to seizure onset **B)** seizure frequency, **C)** seizure duration, **D-G)** seizure severity and **H)** seizure mortality. Ceftriaxone treatment significantly reduced seizure symptoms in CCI mice. ANOVA with Tukey’s multiple comparison test. * = P < 0.05 vs sham. n = 18 for sham, 10 for CCI-vehicle and 10 for CCI-ceftriaxone.

We tested the ability of delayed ceftriaxone treatment to reduce seizure susceptibility in 70dpi CCI mice. Ceftriaxone treatment began at 56dpi through day 70dpi. 2h after the final ceftriaxone administration, mice were challenged with 30mg/kg PTZ and observed for the following 30 minutes. For all severities of seizures there were no significant differences in the latency to seizure onset (Fig 5A), or to the frequency of seizures that occurred over the 30-minute period (Fig 5B). However, there was a 1092% increase in seizure duration in CCI vehicle mice (P = 0.0313) which was reduced to 460% increase in CCI ceftriaxone-treated mice (Fig 5C). There was no statistically significant effect of treatment on low-severity seizures induced by PTZ (Fig 5D and 5E), however ceftriaxone completely blunted the effect of CCI on high severity seizures, reducing both the seizure duration and number of high-intensity seizures. High-intensity seizure duration caused by PTZ was increased by 1015% in CCI vehicle treated mice (P = 0.0389), but not increased in ceftriaxone treated CCI mice (Fig 5F). The number of high-intensity seizures was increased by 990% in vehicle-treated CCI mice (P = 0.0234), but not changed in ceftriaxone-treated CCI mice (Fig 5G). Overall mortality to PTZ was increased from 0% in sham mice to 20% in CCI vehicle mice, and this was reduced to 10% by ceftriaxone treatment in CCI mice (Fig 5H). Ceftriaxone did not improve cognition in the T-maze: sham mice had an alternation score of 85 ± 0.06%, CCI vehicle mice scored 70 ± 0.06% and CCI ceftriaxone mice scored 60 ± 0.1%. We conclude that a delayed 2-week treatment with ceftriaxone can partially prevent the increased susceptibility of CCI mice to PTZ-seizures, and specifically targets the number of severe seizure episodes, and the duration of these seizures.

## Discussion

In this study we demonstrate that mice exposed to a TBI are more susceptible to PTZ-induced seizures than sham mice. This increased susceptibility to PTZ occurs over time, as CCI mice are not more susceptible in the subacute phase after injury, but become more susceptible to PTZ as time from injury increases. This seizure susceptibility is associated with decreased levels of Scl1a2 and Scl1a3 in the cortex and Scl1a2 in the hippocampus at 70dpi. By treating TBI mice in the chronic phase with ceftriaxone for two weeks, we can increase Scl1a2 and Scl1a3 in the cortex and Scl1a2 in the hippocampus and reduce seizure intensity in TBI mice.

Post-traumatic epilepsy is well-documented in human patients, with TBI increasing the risk of epilepsy by three-fold compared to the non-TBI population [33]. The increased risk of seizures after TBI is correlated with the severity of injury with severe TBI resulting in a 17-fold increased risk of seizures, moderate TBI causing a 3-fold increased risk of seizures, and mild TBI causing a 1.5-fold increase in risk. The time since injury is also a variable factor, with the risk associated with mild TBI dissipating within 5 years post-injury, but severe brain trauma remaining a strong risk factor for post-traumatic epilepsy even 10 years post-injury [33]. In preclinical TBI models, post-traumatic epilepsy has been more difficult to document and define, but multiple groups have reported increased spontaneous seizures and seizure-like electrical activity recorded in the brain of FPI rodents [34–36], CCI rodents [19], and in a penetrating brain-injury model [37]. There remains some controversy as to whether the spike/wave discharges being reported in the electrical recordings can really be considered epileptic-like activity as these same discharges can be also observed in control animals, and it is unclear that they are occurring alongside seizure episodes [38]. Despite this controversy, there is clear evidence that TBI makes rodents more susceptible to the chemical seizure agent PTZ. This TBI-induced susceptibility decreases latency to the first seizure, increases seizure frequency, and increases seizure duration. This increased susceptibly has also been reported in FPI mice at 30dpi [39], in juvenile CCI mice at 21dpi [24], and in adult CCI mice at 6- and 9-months post-injury [20].

In the present study we find that adult CCI mice are more susceptible to PTZ—however this is a time-dependent effect as 7dpi CCI mice are not susceptible to 30mg/kg PTZ, but at 70dpi CCI mice have reduced seizure latency, increased seizure frequency and increased seizure duration. Our data adds to the literature that post-traumatic epilepsy is a time-dependent response to injury. This increased seizure susceptibility is not related to tissue loss as we have comparable lesion volumes at 7d and 70dpi. We also find that CCI-induced cognitive impairments are similar at 7dpi and 70dpi.

So, what is causing this time-dependent increase in seizure susceptibility? Excitatory activity is increased in the brains of TBI animals, including increased mossy fiber sprouting [40], increased neuronal hyperexcitability [40], reorganization of excitatory networks [41], reduced inhibitory interneurons [42], elevated glutamate signaling [42] and impaired glutamate recycling [43]. Our data shows that components of the chronic post-traumatic secondary injury cascade must be responsible for the development of seizure susceptibility. In this study we focused on glutamate recycling at the tripartite synapse. Given that GLT-1 is responsible for almost all the glutamate clearance capacity in the rodent brain [13] and reduced levels of GLT1 contributes to reduced seizure threshold and increased lethal seizures [12], we focused on GLT-1 and GLAST in our studies. We found that in the sub-acute phase after CCI, when TBI mice are not more susceptible to PTZ, there is an increase in cortical and hippocampal GLT1 and GLAST. In contrast, we found that in the chronic phase after CCI, when TBI mice are more susceptible to PTZ, there is a normalization and even a decrease in cortical and hippocampal GLT1 and GLAST in the mouse brain. These data are consistent with studies in rat TBI models showing a transient posttraumatic decline in GLT-1 mRNA and protein levels and transporter function that starts within 24 hours of injury and may normalize by 6 weeks after injury [43–46]. The implication of a reduction in these transporters is that glutamate recycling would be impaired at the synapse, leading to a slower clearing of glutamate from excitatory synapses, and potentially increased excitatory activity.

The use of β-lactam antibiotics, including the antibiotic ceftriaxone, to stimulate GLT1 expression through NF-κB expression are well-established [29, 47]. Ceftriaxone increases brain expression and function of GLT1, and ceftriaxone is neuroprotective both *in vitro* and *in vivo* [29]. We found that delayed ceftriaxone treatment (200mg/kg) increased GLT1 mRNA in both the cortex and hippocampus, and GLAST mRNA in the cortex. In addition, ceftriaxone also prevented an increase in seizure duration, specifically reducing the number and duration of high-severity seizures in CCI mice following PTZ. Our data is consistent with the single report on the use of ceftriaxone for the treatment of seizures in TBI, where LFP causes a decrease in GLT-1 protein in the rat at 7d post-injury, and this decrease was reversed by ceftriaxone treatment beginning 30 mins post-injury and continuing for 7 days [44]. This was accompanied by a reduced number of spontaneous seizures detected using a wireless EEG telemetry system [44]. Our study contrasts with this published work in how we demonstrate that delaying ceftriaxone treatment can successfully prevent seizure susceptibility following TBI. Other than glutamate receptors, ceftriaxone may also inhibit seizure formation via protection of inhibitory interneurons after brain trauma [46], which would improve the excitatory/inhibitory balance after TBI. It is important to note that ceftriaxone can protect against seizures in naïve/uninjured mice with multi-day ceftriaxone treatment reducing PTZ-induced seizure duration and severity [48–51]. This demonstrates that the seizure-reducing effects of ceftriaxone are not specific for TBI, however given the intractable nature of PTE [8, 9], any evidence for the benefit of anti-epileptic medications can help guide therapeutic interventions.

There are a number of limitations to our study. We have used a single model of chemical-induced seizures in our study, and other rodent epilepsy models such as pilocarpine, electroconvulsive shock, or monitoring of spontaneously generated seizures using EEG recordings may better help characterize the beneficial effects of ceftriaxone. We used a two-week dosing regimen starting at 55dpi and continuing until 2h prior to the final PTZ trial. We did not test if a single ceftriaxone injection at 2h prior to PTZ is sufficient to prevent PTZ-induced seizures in 70dpi CCI mice. However, given that the proposed mechanism of action of ceftriaxone is via glutamate transporters it is unlikely that a single dose would be effective. We also did not test if ceftriaxone could reduce seizures at 7dpi, but as ceftriaxone can reduce PTZ-induced seizures in non-injured mice [48–51], and as many epilepsy drugs can reduce acute but not chronic PTE seizures, [52] we expect ceftriaxone to reduce PTZ-induced seizures in 7dpi mice. Another limitation of our study design is that we measured glutamate transporter mRNA at the conclusion of our study, which included the PTZ-challenge. In our second study, the mice were also exposed to daily handling and intraperitoneal injection, which could increase stress in the test mice and modify our results compared to our first study.

In conclusion, our study confirms previous findings that severe TBI increases PTZ-induced seizure susceptibility, however we demonstrate that susceptibility is time-dependent and occurs in the chronic, but not sub-acute phase after CCI. We also show for the first time that delayed treatment with ceftriaxone can protect the brain against epileptic activity, specifically by reducing PTZ-induced seizure intensity and seizure duration in CCI mice. Due to the ongoing debate regarding antibiotic resistance, the use of ceftriaxone or other antibiotics to achieve this goal remains questionable–however, our data provides support for the use of drugs targeting glutamate transporters for the treatment of seizures after TBI.

## Supporting information

S1 Data (XLSX)
